# Congenital Granular Cell Tumor – A Rare Entity

**Published:** 2015-04-01

**Authors:** Monal Yuwanati, Shubhangi Mhaske, Ashok Mhaske

**Affiliations:** 1 Dept of Oral Pathology and Microbiology, Peoples Dental academy, Bhopal; 2 Dept of General Surgery, Peoples College of Medical Sciences, Bhanpur bypass road, Bhopal

**Keywords:** Neumann’s tumor, Congenital granular cell Myoblastoma, Granular cell fibroblastoma, Congenital granular cell epulis, Congenital epulis

## Abstract

Congenital granular cell tumor is a rare benign neoplastic growth affecting the gingival mucosa of neonates. Prenatal ultrasound diagnosis has recently come to focus and in spite of several reports on immune-histochemical and other advanced marker studies, the cause and origin of the lesion remains debatable till date. Review of literature on prenatal diagnosis and histopathology along with immunohistochemistry is discussed.

**Introduction:**

Congenital Epulis (CE), or congenital granular cell tumour (CGCT), is a congenital benign rare tumour of the newborn. The original first description of the lesion was dated in 1871 by Neumann [1]; approximately 250 cases have been reported since then [2]. Other terminologies for CGCT described in literature are congenital epulis, granular cell rabdomyoma, congenital myoblastoma, or Neumann’s tumour. The term 'epulis' has been used, but this simply means swelling on the gingival. It was suggested to be discontinued and congenital granular cell tumour to be used in the literature. Currently “congenital epulis of newborn” is universally accepted and frequently employed terminology in the literature [3]. 

The congenital gingival granular cell tumour is most frequently seen on the alveolar median ridge of the maxilla than alveolar ridge of mandible (1:3). Incisor-canine area is commonly affected. It has a female preponderance. Incidence rate of the CGCT is unknown but suggested to be 6 per million [4]. The clinical diagnosis of CGCT is almost obligatory when a solitary tumour of the gingiva is present at birth. The presentation is unique at birth as fibrous mass arising from the gingival mucosa or alveolar ridge of the maxilla or mandible. CGCT appears sporadically and has no familial tendency [5]. It most commonly originates from the anterior alveolar ridge, with the maxilla being involved twice as much as the mandible [6], though the lesion can arise from the tongue, palate, skin, the subcutaneous tissue, skeleton muscles, the vocal chords, and in small number cases from the rest of the body. This pedicular tumor has a smooth or lobular surface and a firm, rubbery consistency [7]. Ultrasonography confirms diagnosis as early as at 26 weeks of gestation or in third trimester of pregnancy [8]. The histogenesis of CGCT has been long debatable, as various authors suggested different source of origin of the tumour. The proposed source of origin includes undifferentiated mesenchymal cells [9], odontogenic epithelial, pericytic, and fibroblastic, histiocytes, nerve-related, smooth muscle, and primitive mesenchymal cells [10]. The histogenesis remains still unclear despite several studies. Though spontaneous regression of the lesion has been reported [11, 12], surgical excision is the only valuable therapeutic option which can be beneficial to both mother and newborn. Recurrence or malignant transformation is not yet mentioned in available literature.

**Site**

Typically, a single tumour is present (90%), ranging in size from several millimetres to several centimetres [13]. Multiple lesions (10%) has been reported involving either or both jaws and have been described with associated abnormalities of the nasal bridges and septum [13-15]. Classically, it arises from the pre-incisor-canine area [11]. Literature survey has suggested that maxilla as most common site of involvement [Fig. 1].

**Figure:**
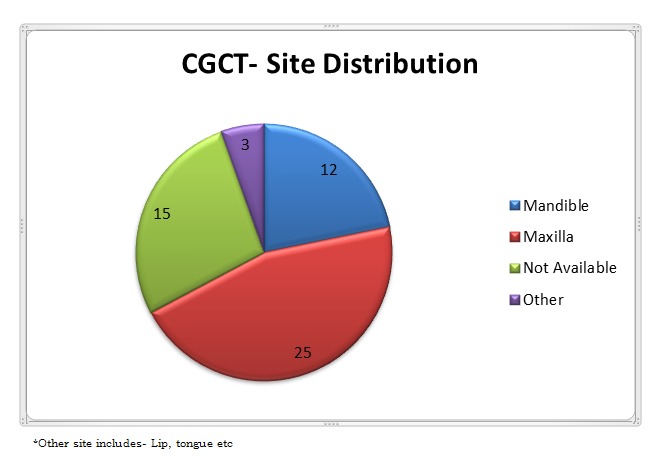
Figure 1: Site distribution

**Prenatal Diagnosis**

Prenatal imaging of congenital lesions of oral cavity is possible by ultrasound (USG) or Magnetic resonance Imaging (MRI) and can be helpful in planning the delivery as well as postnatally in demonstrating the congenital lesion and treatment planning. With the help of Ultrasonography or MRI, tumor mass can be identified, mainly in last weeks of pregnancy [16]. Ultrasonography or even MRI can only show the presence of tumor mass suggestive of epulis, but the diagnosis could not be conclusive. However, few studies indicated in past has state successful prenatal diagnosis of congenital epulis using ultrasonography [5]. Postnatally, it may interfere with feeding [14], or respiration. Prenatal diagnosis of CGCT can aid in counselling the parents as to the nature and treatment of the abnormality, as well as the potential risk of airway obstruction and intraoperative complications. Prenatal ultrasound investigation is important, since large tumours may interfere with vaginal delivery and a caesarean may be required [5, 11, 13]. The discovery of a tumor mass on a foetus may help the intervention planning as well as to prepare the parents mentally for eventual measures that may occur in the newborn. Prenatal diagnosis of the lesion on ultrasonography was reported in the literature as early as 26 to 38 weeks [Table 1] which is mostly coincide with third trimester of the pregnancy. It’s very striking that lesion is not detectable till that period, reason for this are unclear. Kusukawa et al. have demonstrated a positive correlation between tumor size and estimated fetal weight [17].

**Pathogenesis**

It has unique female preponderance [Table 1, 2 and Fig. 2]. Based on the theory supported by the experimental production of CGCT in a mouse following injection of endogenous hormone, this characteristic finding was suggested to be attributable to presence of endogenous hormones and sudden regression of mass after birth [14]. This was subsequently failed proved as there was no detectable presence of these endogenous hormone receptors with the lesion tissue. Trauma, due to finger sucking in utero, was suggested not considered significant [11, 12, 18]. Recently, it was suggested that it may be a local metabolic or reactive change [19]. 

Table 1 Supplementary PDF file supplied by authors.

Table 2 Supplementary PDF file supplied by authors.

**Figure:**
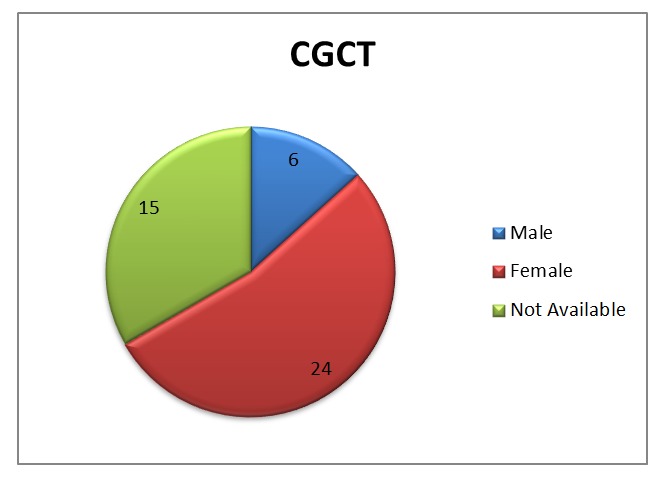
Figure 2: Gender distribution

It most commonly presents as an isolated finding with no known association with other congenital anomalies though reported to be associated with neurofibromatosis, polydactyly, binder syndrome, congenital goiter [20], and bilateral transverse facial cleft [21]. Rarely, shows association with absence of underlying tooth/germ. Majority prenatal associated complications reported are polyhydramnios, obstructed deglutition of amniotic fluid whereas postnatally causes midface hypoplasia, incisor hypoplasia, severe feeding and respiratory/aesthetic problems. As there is potential risk of neonatal respiratory distress, it was suggested that prenatal diagnosis becomes prerequisite for uneventful pregnancy. Ultrasonography is crucial in such cases.

**Differential Diagnosis**

Diagnosis of congenital epulis can usually be made on characteristic clinical findings. Clinical differential diagnosis (D/D) is broad based and is depends upon site of involvement, size velocity of growth and possible accompanying lesion or other developmental anomalies like Epstein pearls, granular cell tumour, vascular malformations and neuroectodermal tumours of infancy.

Predominance of female patients, tumor location on maxillary anterior region, presence at birth and absence of growth potential and the possibility of spontaneous remission without any intervention could rule out majority of the D/D. Various lesions that can be considered prenatally are congenital malformations, dermoid cyst, haemangioma, lymphatic malformations, melanotic pigmentation neuroectodermal tumours of infancy, rhabdomyosarcoma, granular cell tumor, oral teratoma-epignathus as well as other possible diagnoses such as fibroma, lipoma, leiomyoma, rhabdomyoma, peripheral giant cell granuloma, pyogenic granuloma, neurofibroma, myxoma, hemangioma, lymphangioma, and alveolar lymphangioma [10]. The location and sonographic appearance aid in differentiating these masses. Teratomas often contain calcifications. Hemangiomas arise externally from subcutaneous tissues and may be solid or cystic in appearance. The accurate diagnosis has been further complicated by its similarity, histologically, with the Granular cell tumor/ myoblastoma (GCT), which occurs in adults at a number of intraoral sites, such as tongue. CGCT can be separated from Granular cell tumor (GCT) by location, patients age, absence of cytoplasmic hyaline granules, solid growth pattern, pericytic proliferation, attenuated overlying epithelium, negativity of lesion tissue to S- 100 [Table 2, 3 and Fig. 3]. d. 

**Figure:**
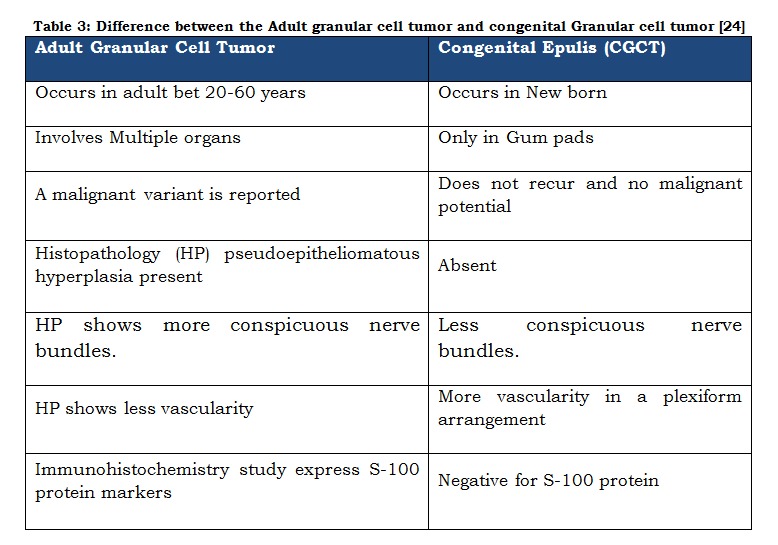
Table 3: Difference between the Adult granular cell tumor and congenital Granular cell tumor [24]

**Figure:**
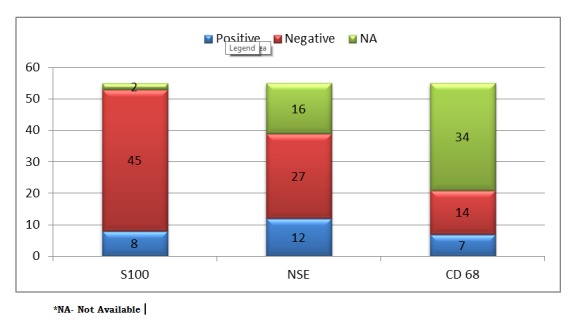
Figure 3: Immunohistochemistry marker used in congenital Granular cell tumor cases

**Immunohistochemistry**

The immunohistochemistry of the tumor is diverse in newborns and adults. Congenital granular cell tumor/epulis is S-100 negative and does not show differentiation to specific cell type [4, 19]. Possible histological origins of the epulis may include epithelial and undifferentiated mesenchymal cells, pericytes, fibroblasts, smooth muscle, nerve-related cells, and myofibroblasts [22]. It has been suggested to be a nonneoplastic, degenerative, or reactive lesion [23].

**Histopathology**

Characteristic histological findings shown by these congenital epulides include large round cells with granular, eosinophilic cytoplasm and small eccentric nuclei and a delicate fibrovascular network separating the cells. Histopathologically, it known that congenital epulis consist of granular cell and is similar to the adult granular cell tumor, but there are some differences such as pseudo-epithelialomatous hyperplasia, lesser vascularity, more conspicuous nerve bundles than congenital epulis [Table-3] [24]. Pseudoepitheliomatous hyperplasia of the oral mucosa on the tumor surface is seen in 50 % of adult GCT cases. but is usually absent in CGCT cases. Spindle cell variant of granular cell Cases with accompanying pseudo-epitheliomatous hyperplasia or ulceration may mimic squamous cell carcinoma [25]. 

**Treatment**

Treatment is with surgical excision; with few cases of spontaneous regression has been reported [26]. After surgical removal, no recurrence or malignant changes have been reported even after incomplete excision, and spontaneous regression may occur [12]. Any Delay in operation can lead to airway obstruction and feeding difficulty. The tumour should be removed during the immediate postnatal period and is usually achieved without serious consequences. If the lesion is smaller conservative approach can be taken to avoid unwanted surgery. Prognosis in CGCT is good as there no reported case of recurrence even after the incomplete removal of lesion [10]. 

**Conclusion**

The main areas of controversy surrounding the congenital epulis remain with the exact aetiology, growth, and progression of the lesion in the foetus. The lesion still poses scope for further research in exact etiopathogenesis. Prenatal Diagnosis is an important aspect to be considered specifically in this entity. Treatment planning should be based on the prenatal diagnosis, size of the lesion etc. 

## Footnotes

**Source of Support:** Nil

**Conflict of Interest:** None

