# Supplementary material for: Congenital Granular Cell Tumor – A Rare Entity
Source: J Neonatal Surg. 2015 Apr 1;4(2):17. (PMC4447470)
Supplement: Table 1 — Supplementary PDF file supplied by authors. [file jns-4-17-s1.pdf]

Table 1- Immunohistochemistry profile of CGCT cases cited literature*

SR	Author	Sex	Site	Diagnosis	Immunohistochemistry	S-100	NSE	CD 68
1.	Nakata M, et al. [4]	Female=1	Alveolar ridge of the mandible	CGCT	Negative on Periodic acid-Schiff (PAS). Partially positive for S-100 protein Negative for neuron specific enolase (NSE).	+	-	NA
2.	Charrier JB [27]	Male=1	mandibular ridge	CGCT	Positive S-100	+	NA	NA
3.	Adeyemi Bukola, et al. [28]	Female=1	Anterior mandibular ridge	CGCT	Strongly Positive for Vimentin and NSE focally and weakly for S-100 Negative for Desmin and Cytokeratin AE1/AE3	+	+	NA
4.	Hasanov A, et al. [29]	Female=1	Mandibular alveolus.	CGCT	No PAS positivity in the tumor. S100, CD68, desmin, MSA, myogenin, SMA and NSE were also negative.	-	-	NA
5.	Pellicano M, et al. [30]	Female=1	Alveolar process of the maxilla	CGCT	Periodic acid-Schiff (PAS)-negative but positive to the S100 protein	+	NA	NA
6.	Damm DD, et al. [22]	Female=1	Gingiva of the anterior maxilla	CGCT	negative for S100 protein immunostain	-	NA	NA
7.	Pinto JGS, et al. [31]	Female=1	left side of the mandibular alveolar ridge	CGCT	Negative for protein S100	-	NA	NA
8.	Sigdel B, et al. [32]	Female=1	maxillary alveolar ridge	CGCT	The tumor cells were negative for Periodic acid-Schiff (PAS) stain. Immunohistochemical evaluation was as follows: Vimentin: Strongly positive (fig. 6), NSE: Positive, CD68: Positive. Cytokeratin, Desmin, CEA, ER, PR, S-100 protein: Negative.	-	+	+
9.	Oslon JL, et al. [33]	Female=1	Left maxillary alveolar ridge	CGCT	Negative for S-100 staining	-	NA	NA
10.	Childers EL, et al. [34]	Nine lesions were in females; 1 case did not designate patient sex=10	6 on the maxilla, 2 on the mandible, 1 on the designated maxillary lip, and 1 unknown. The cases included a patient with 2 lesions: 1 on mandibular and 1 on maxillary alveolar ridges	CGCT	All cases were negative for S-100 protein, CD68, CD34, CD31, keratins, desmin, calponin, and smooth muscle actin.	-	-	-
11.	Tellado MG, et al. [35]	Case 1- Male=1<br>Case 2- Female=1	canine region right maxilla<br>right maxillary region	CGCT	The S100 was negative.	-	NA	NA

12.	Abo-Hager EA, et al. [36]	six cases of congenital granular cell epulis<br>All cases Female=6	All cases were from the anterior maxillary alveolar ridge	CGCT	All tumors were positive for NSE, granular cells showed a cytoplasmic immunostaining for NSE)<br>The granular cells in All cases - positive immunostaining for CD68.<br>The interstitial cells (five) immunopositivity for CD99.<br>Granular and interstitial cells in all cases - negative with the remaining antibodies (Mesothelin, Inhibin-α, GFAP, Dystrophin, NGFR/p75 and TLR1).	-	+	NA
13.	Rehman MU, et al [37]	Female =1	Gingiva of maxilla	CGCT	Negative for immunostaining by S100, CKAE1/AE3, actin, and desmin.<br>Tumor cells were reactive for CD68, a macrophage marker	-	NA	+
14.	Wittebole A, et al. [15]	Female=1	left incisor region of the mandible	CGCT	Negative for S-100 protein	-	NA	NA
15.	Lapid O, et al. [38]	Female=1	gingiva of the left maxilla	CGCT	Positively with Periodic acid-Schiff reagent and the staining was not removed by pretreatment with diastase.<br>The Diffusely and strongly positive for vimentin, and negative for S100-protein, actin, desmin, laminin, keratin, estrogen, and progesterone receptors	-	NA	NA
16.	Dhingra M, et al [39]	Female=1	Gingiva of maxilla	CGCT	Diastase resistant periodic acid Schiff positive.<br>Positive for vimentin negative for S100, desmin and actin.	-	NA	NA
17.	Okina T, et al. [40]	7-day-old female=1<br><br>37 day-old male infant.=1	Gingiva of the lower jaw<br>on the alveolus of the lower deciduous incisors,	CGCT	Weakly PAS positive	NA	NA	NA
18.	Anderson AJ, et al. [41]	Female=1	Anterior maxillary alveolus	CGCT	Positive for actin and myosin but not S100	-	NA	NA
19.	Dzieniecka M, et al. [42]	Female=1	Anterior ridge of the maxilla.	CGCT	Negative for S-100	-	NA	NA
20.	Leocata P, et al. ~~[42]~~ [18]	Male=1	Anterior maxillary ridge	CGCT	Positivity was observed only for vimentin, whereas desmin appeared focally and moderately positive.<br>Alpha-fetoprotein; carcino-embryonic antigen (CEA); macrophage marker;	-	-	-

					lysozyme; CD-68; NSE; S-100 protein; muscle-specific actin; leukocyte common antigen; estrogen receptors; progesterone receptors were negative.			
21.	Kayiran SM, et al. [43]	Female=1	anterior ventral surface of the tongue	CGCT	S-100 did not show any positivity, and CD34 staining emphasized a prominent vascularity.	-	NA	NA
22.	Prigkos AC, at al. [44]	Male=1	Mandibular alveolar ridge	CGCT	Intensely positive for vimentin and NSE<br>Negative for pan-keratin, S-100 protein, carcinoembryonic antigen (CEA), glial fibrilliary acidic protein (GFAP), smooth muscle actin, muscle specific actin (HHF35), desmin, CD68, and CD31.<br>Smooth muscle actin, muscle specific actin (HHF35).<br>CD31 revealed a complex network of vascular channel	-	+	-
23.	Inan M, et al. [45]	Male=1	Right Maxillary Alveolus	CGCT	Positive for Vimentin<br>Negative for S-100, Desmin, NSE, Glial Fibrillary Acidic Protein.	-	-	NA
24.	Filie AC, et al. [46]	Out 10 case, 3 Congenital granular cell tumors.	NA	CGCT	All of the GCTs were negative for keratin, smooth muscle actin, muscle-specific actin, desmin, CD57, CD15, and MAC387 Congenital GCTs, on the other hand, were negative for S100 and NSE but positive for A1AT, CD68, and vimentin.	-	-	+
25.	Kaiserling E, et al. [47]	Four Granular cell tumor- Two congenital epulis	NA	CGCT	CGCT was positive for CD68+, Ki-M1P+, lysozyme-, vimentin+, fibronectin+, laminin+, lectin PHAE+, and lectin WGA+<br>1 Congenital epulis was weakly positive for S-100 (other was negative)	+<br>-	NA	+<br>+
26.	Vered M, et al. [19]	Five CGCEs cases	NA	CGCT	Positivity of these cells to vimentin, NKI/C3, and PGP9.5.<br><br>Non-reactive for S-100, NGFR/p75, and inhibin-alpha	-	NA	NA
27.	Takahashi H, et al. [48]	Three cases	NA	CGCT	Neoplastic cell- Positive for Neuron-specific enolase (NSE) and vimentin. However, all other reactions were negative.<br>Interstitial cell strong S-100 protein-, cytokeratin-, vimentin-, and NSE-immunostainings, and these cells are consistent with neuroendocrine nature.	-	+	NA

28.	Luis M, et al. [49]	Two cases	NA	CGCT	Positive immunostaining was found for S-100 protein and neuron-specific enolase in all the cases of myoblastoma, and for vimentin and carcinoembryonic antigen in some cases. No immunoreactivity was observed for any of the other 13 antibodies used in congenital epulis.	+	-	-
29.	Messina M, et al. [50]	Male	Lateral to oral cavity close to tongue	CGCT	NA	No	NA	
		Female	Right side Maxillary alveolar ridge.	CGCT	NA	No	NA	
30.	Majid ZA, et al. [51]	NA	NA	CGCT	NA	NA	NA	NA
31.	Hoyme HE, et al. [52]	NA	NA	CGCT	NA	NA	NA	NA
32.	Raissaki MT, et al. [53]	Female	Maxillary alveolar ridge	CGCT	NA	NA	NA	NA
33.	Taylor P, et al. [54]	Female	Midline attached to gingival of maxilla	CGCT	NA	NA	NA	NA
34.	McMahon MG, et al. [55]	Female	Anterior maxilla	CGCT	NA	+	NA	NA
35.	Pellicano M, et al. [30]	Female	Alveolar process of the maxilla	CGCT	NA	NA	NA	NA
36.	Lopez de Lacalle JM, et al [56]	NA	NA	CGCT	NA	NA	NA	NA
37.	Szlachetka K, et al. [57]	Female	Mandible	CGCT	NA	NA	NA	NA
38.	Bornstein E, et al. [58]	Female	Anterior to tongue- left mandibular ridge.	CGCT	NA	NA	NA	NA
39.	Koch BL, et al [11]	Female	Anterior Maxillary alveolar ridge<br>Anterior Mandibular ridge	CGCT	NA	NA	NA	NA
40.	JM Su, et al [22]	Female	right side of the midline and was found to be attached to the gingival of the anterior alveolar ridge of the mandible	CGCT	NA	NA	NA	NA
41.	Lu W, et al.	Female	Solitary Mandibular 2.5	CGCT	NA	NA	NA	NA

	[59]		x 2 x 2 cm					
42.	Nakata M, et al. [4]	Female	alveolar ridge of the mandible	CGCT	NA	NA	NA	NA
43.	Charrier, JB. [27]	Male	mandibular ridge	CGCT	NA	NA	NA	NA
44.	Shaw L, et al. [60]	Female	alveolar ridge of the mandible	CGCT	NA	NA	NA	NA
45.	Thoma V, et al. [61]	-	-	CGCT	NA	NA	NA	NA
46.	Yvonne Nam RT. [62]	-	-	CGCT	NA	NA	NA	NA
47.	Meizner I, et al. [63]	-	-	CGCT	NA	NA	NA	NA
48.	Hulett RL, et al. [64]	NA	NA	CGCT	NA	NA	NA	NA
49.	Fister P, et al. [6]	Female	Maxillary Alveolus left to midline	CGCT	NA	NA	NA	NA
50.	Song WS, et al. [16]	Female	Mandibular alveolar ridge	CGCT	NA	NA	NA	NA

*** (Total = 55 cases in which information about IHC was retrieved)**
