# Supplementary material for: Congenital Granular Cell Tumor – A Rare Entity
Source: J Neonatal Surg. 2015 Apr 1;4(2):17. (PMC4447470)
Supplement: Table 2 — Supplementary PDF file supplied by authors. [file jns-4-17-s2.pdf]

Table 2: Cited report of CGCT diagnosed prenatally on ultrasound Sonography

SR No	Author	Sex	Site	Prenatal Diagnosis	Histological diagnosis	IHC markers	Follow up	Recurrence
1.	Fister P, et al. [10]	Female	Maxillary Alveolus left to midline	NA	CGCT	No	2 years	No recurrence even after incomplete resection.
2.	Messina M, et al [50]	Male	Lateral to oral cavity close to tongue	36th gestational week [Doppler echocardiography 3D/4D]	CGCT	No	NA	NA
		Female	Right side Maxillary alveolar ridge.	Showed no lesion or associated anomalies	CGCT	No	NA	NA
3.	Majid ZA, et al [51]	NA	NA	NA	NA	NA	NA	NA
4.	Hoyme HE, et al [52]	NA	NA	NA	NA	NA	NA	NA
5	Raissaki, MT, et al. [53]	Female	Maxillary alveolar ridge	24 antenatal week [Unremarkable]	CGCT	NA	NA	NA
6	McGuire TP, et al. [54]	Female	Midline attached to gingival of maxilla	29th weeks [No abnormality]	CGCT	NA	NA	NA

7	McMahon MG, et al. [55]	Female	Anterior maxilla	37 week	CGCT	S-100	NA	NA
8.	Pellicano M, et al. [20]	Female	Alveolar process of the maxilla	12 & 22 Weeks [no abnormality detected]<br><br>31[mass detected]	CGCT	NA	NA	NA
9	Lopez de Lacalle JM, et al [56]	NA	NA	NA	NA	NA	NA	NA
10	Song WS, et al. [16]	Female	Mandibular alveolar ridge	36 weeks	CGCT	NA	NA	NA
11	Szlachetka K, et al [57]	Female	Mandible	24 and 28 week [No Abnormality evident]<br><br>37 week [well circumscribed]	CGCT	NA	NA	NA
12	Bornstein E,et al [58]	Female	Anterior to tongue- left mandibular ridge.	15 and 22 wks [No Abnormality detected]<br><br>32 wks [Protruding mass left mandibular alveolar ridge]	CGCT	NA	NA	NA
13	Koch BL, et al. [11]	Female	Anterior Maxillary alveolar ridge<br><br>Anterior Mandibular ridge	38 weeks	CGCT	NA	NA	NA
14	JM Su, et al. [21]	Female	Right side of the midline and was	32 week	CGCT	NA	NA	NA

			found<br><br>to be attached to the gingival of the anterior alveolar ridge<br><br>of the mandible					
15	Lu W, et al.<br><br>[59]	Female	Solitary Mandibular; 2.5 x 2 x 2 cm	33 weeks of<br><br>pregnancy [without abnormality]	CGCT	NA	NA	NA
16	Nakata M, et al. [4]	Female	Alveolar ridge of the mandible	26 weeks of gestation	CGCT	positive for S-100 but negative for NSE	No recurrence	1 year
18	Charrier JB, et al. [27]	Male	Mandibular ridge	38 week of gestation [airway obstructions]	CGCT	Positive S-100	NA	NA
19	Shaw L, et al. [60]	Female	Alveolar ridge of the mandible	22weeks' gestation [No detection] 27 weeks' gestation [mass detected] 29 weeks [mass detection with increase in size]	CGCT	NA	NA	NA
20.	Thoma V, et al. [61]	-	-	32nd week of pregnancy	CGCT-	-	-	-
21	Yvonne Nam RT. [62]	-	-	39 weeks gestation	-	-	-	-

				[intraoral abnormality]<br><br>20 weeks, 6 days demonstrated no abnormalities.				
23	Meizner I, et al [63]	-	-	15 and 22 weeks of gestation [No abnormality]<br><br>29 weeks [mass detected]	-	-	-	-
24	Hulett RL, et al. [64]	NA	NA	NA	NA	-	-	-

*** NA- Information Not Available**
